# Association of grip strength and comorbidities with all-cause mortality in the older hypertensive adults

**DOI:** 10.3389/fpubh.2023.1162425

**Published:** 2023-06-28

**Authors:** Ying Wang, Tianyu Meng, Wei Yang, Miaojia Yan, Xianming Su, Xiaohong Wang, Lihong Chen, Yanping Ren

**Affiliations:** ^1^International Medical Center, The First Affiliated Hospital of Xi'an Jiaotong University, Xi’an, Shaanxi, China; ^2^Department of Geriatrics, The First Affiliated Hospital of Xi'an Jiaotong University, Xi’an, Shaanxi, China; ^3^Department of Medical Administration, The First Affiliated Hospital of Xi 'an Jiaotong University, Xi 'an, China

**Keywords:** handgrip strength, comorbidity, hypertension, older adults, mortality

## Abstract

**Background:**

With growing concerns about global population aging, comorbidity, and disability have emerged as key variables that influence the health of the older adults in terms of disease and function. This study sought to examine the impact of comorbidity and impairment using disease and functional status indicators of all-cause mortality in the older adults. Hypertension, which was chosen as the indicator chosen for disease, has the greatest prevalence in the older population. A total of 15 self-reported chronic conditions were added as indicators of comorbidity, and grip strength was chosen as a measure of functional status. The study also evaluated the association between grip strength and comorbidity, as well as its consequences on all-cause death and survival in a hypertensive senior population.

**Methods:**

We chose a total of 2,990 hypertensive participants aged ≥60 years whose data for grip strength were collected in the National Health and Nutrition Examination Survey conducted between 2011 and 2014. The association of all-cause death with grip strength and comorbidity was examined using a Cox proportional hazard regression model. The interaction between comorbidity and all-cause mortality, as well as its association with grip strength, was also examined.

**Results:**

The hazard ratio [95% confidence intervals (CIs)] for all-cause mortality in the highest grip strength tertile was 0.266 (0.168–0.419), compared to the lowest grip strength tertile. The all-cause mortality decreased with an increase in the number of co-morbidities [2.677 (1.557–4.603) in the group with ≥3 chronic diseases]. The weighted generalized model revealed a negative correlation between grip strength and comorbidities in more than three groups after accounting for all possible variables (β = −2.219, −3.178 ~ −1.260, *p* < 0.001). The risk of mortality reduced with increasing grip strength in patients with ≥3 comorbidities (*p*-value for trend <0.05), but no meaningful difference was found in the interaction between comorbidities and grip strength (*p*-value for interaction >0.05).

**Conclusion:**

In older hypertension patients, grip strength and comorbidities were correlated with all-cause death, and there was a negative correlation between grip strength and comorbidities. Higher grip strength was associated with fewer fatalities in patients with ≥3 comorbidities, suggesting that functional exercise can improve the prognosis of comorbidities.

## 1. Introduction

As global aging accelerates and average life expectancy rises, disability and comorbidities have grown in importance, raising concerns for global health care (1). The US Department of Health and Human Services (DHHS) defines comorbidity as a medical condition involving two or more conditions that have each lasted more than a year. In developed countries, the prevalence of comorbidity in senior people aged 65 and older is >60% (64.75% in the US), and more than half of the older adults have more than three chronic conditions (2, 3). Comorbidities in older adults patients result in more complex diagnoses and treatments.

The number of adults living with cardiovascular disease (CVD) in old age is increasing with the aging population, and so is the number of older adults susceptible to CVD as a normal physiological change of aging. Reportedly, more than 70% of adults have CVD by the age of 70, and more than two-thirds have non-CVD comorbidities (4). Comorbidities are common in the older adults, particularly those having CVD. Further, the functions of human body organs gradually slow down with age, due to a variety of chronic diseases that are prone to geriatric syndrome, which not only reduces independent living ability but can even render people disabled. According to the Centers for Disease Control and Prevention Disability and Health Database, the disability rate among people aged 65 and older in the United States reached 42.3% in 2017. The current clinical care regimen is largely based on clinical practice guidelines for disease diagnosis and treatment, as well as management and decision-making. To evaluate the value of a specific treatment, the majority of randomized controlled trials (RCTs) enroll a reasonably homogeneous sample for a particular disease. Patients with multiple diseases are frequently excluded on purpose. Studies involving cardiovascular RCTs majorly focus on cardiovascular adverse events, which may be less helpful than studies that evaluate the overall benefit of treatment in older patients, such as loss of physical and cognitive function or health-related lifetime treatment (5).

Grip strength is a simple, non-invasive measure of muscle strength and function that has been linked to a variety of age-related health problems, including disability. Reduced grip strength has been shown to predict premature death and disability (6). Therefore, grip strength is a clinically viable tool for screening health status during the process of aging. In several prospective studies, grip strength was found to be inversely related to CVD, etiology-specific mortality, and all-cause mortality outcomes (7–9). In a cross-sectional study of Chinese individuals over the age of 50, reduced grip strength was significantly linked to a higher incidence of 12 and 8 chronic diseases in men and women, respectively (10). The results of the Kara-Age study in Germany showed an independent negative association between grip strength and morbidity in older women (11). In most of these studies, the association of grip strength with comorbidities and all-cause mortality in CVDs remains unclear. Given the high prevalence of hypertension in the global population, the primary goal of our cross-sectional study was to evaluate the association of grip strength with comorbidities and all-cause mortality in a hypertensive population. We also wanted to investigate if the presence of comorbidity in individuals with different grip strength levels affects the survival outcome, by studying the relationship between function and disease in older people with CVDs.

## 2. Materials and methods

### 2.1. Study population

The data for this study was provided by the National Health and Nutrition Examination Survey (NHANES), a periodic cross-sectional survey of the U.S. population. The survey used a complex, multi-stage probability sampling method to obtain a nationally representative sample of about 5,000 people per year. The sample design consisted of four stages of multi-year, hierarchical, and clustering samples, with data being released every 2 years. The survey was conducted by the National Centers for Health’s Board of Approval Statistics and Institutional Review. Informed consent was obtained from all study participants (12, 13).

**Figure 1:**
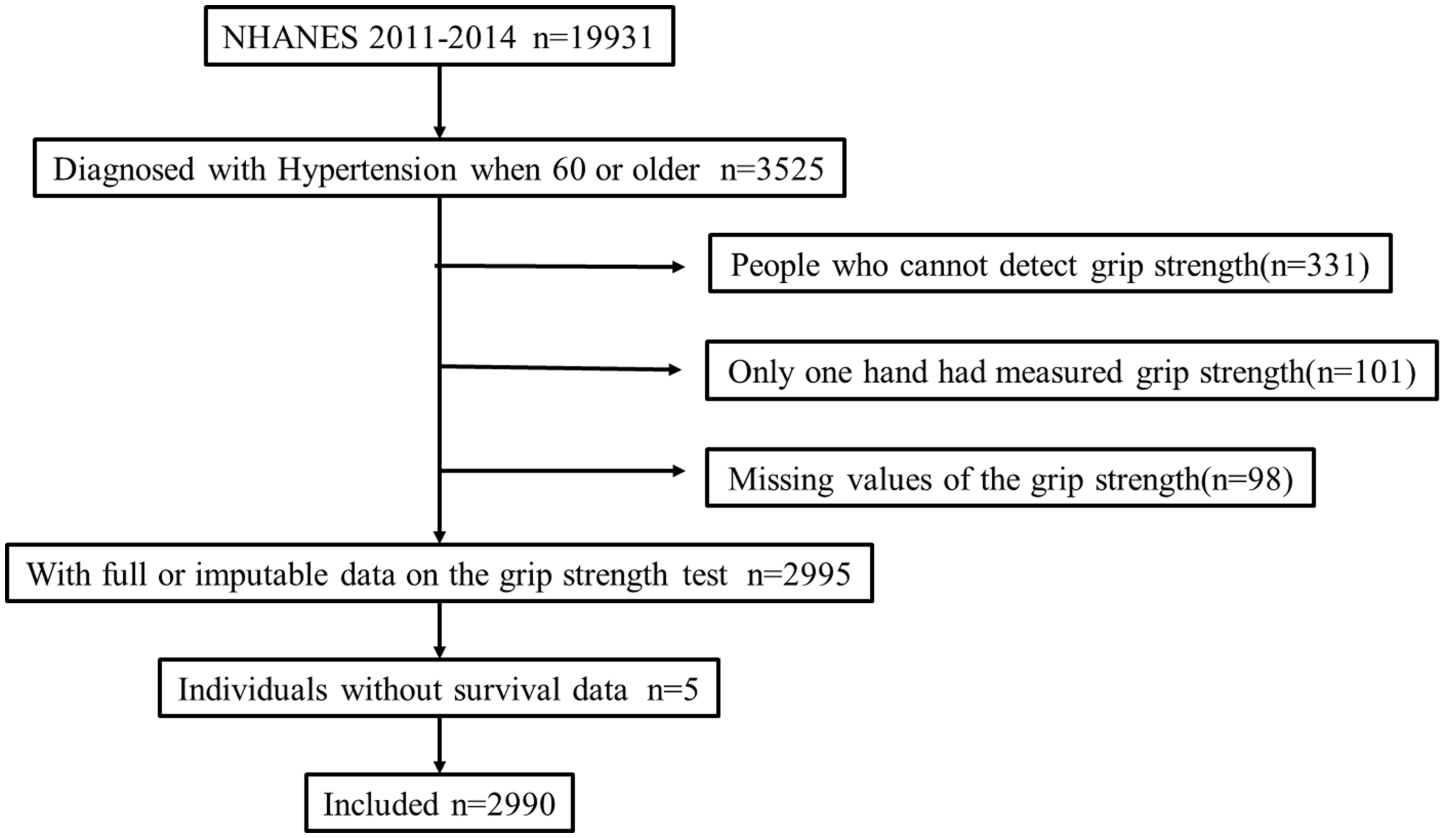
Flowchart of the research sample selection process.

A flow chart of the study sample selection process is displayed in Figure 1. Our study included a total of 19,931 participants. Of these, we chose participants with hypertension who were 60 years or older, while excluding those under 60. The final sample group for analysis consisted of 3,525 hypertension patients aged ≥60 years. Those aged ≥60 years and diagnosed with hypertension were eligible for this study if they met any of the following criteria: (1) In response to the interview question, “Have you ever been told by a doctor or other healthcare provider that you have high blood pressure?” The answer was “yes;” (2) In response to the interviewer’s question, “Do you use blood pressure medication?” The answer was “yes”; and (3) Patients whose mean blood pressure was >140/90 mm Hg and had their blood pressure measured at least three times on different days.

### 2.2. Grip strength

Grip strength was measured using a dynamometer (Takei Digital Grip Strength Dynamometer, Model T.K.K.5401; Takei Scientific Instruments Co. Ltd. Niigata, Japan). The participants were instructed to stand and use one hand to exert as much force as they could on the dynamometer. Each hand was tested three times, with a gap of 60 s elapsing between measurements on the same hand. Grip strength (in kg) was calculated as the mean of left and right hands (7). A total of 2,995 participants were finally selected after excluding 98 participants whose values were missing values, 331 participants whose grip strengths could not be measured, and 101 participants who only had one hand grip strength. Grip strength was considered to be a continuous variable, which was divided into three groups (Tertile 1: <23.4 kg, Tertile 2: 23.4–31.9 kg), and Tertile 3: >31.9 kg) based on the grip strength tertile 23.4 kg and 31.9 kg.

### 2.3. Physical comorbidities

We used data on 15 self-reported chronic diseases, including angina, arthritis, asthma, chronic bronchitis, congestive heart failure, coronary heart disease, diabetes, emphysema, gout, liver disease, myocardial infarction, stroke, thyroid disease, and kidney disease. Participants were classified as having various comorbidities if they answered “yes” to this question. The total comorbidities for each participant were individually calculated and divided into four groups namely, “none,” “one,” “two,” and “three or more.”

### 2.4. Covariates

The covariates included age, sex, race (white, Mexican American, black, and others), an education level (above high school, high school graduate/General Educational Development (GED), and below high school), citizenship status (married/living with a partner, single, divorced/separated, and widowed), poverty income ratio (PIR), smoking status (never, former, and present), alcohol consumption (<12 drinks per year or ≥ 12 drinks per year), health insurance status (insured and uninsured), body mass index (BMI), and waist circumference (cm).

### 2.5. Outcome

All-cause mortality, as determined by the National Death Index (NDI), was considered the primary outcome variable. NDI is one of the reliable resources that is widely used in death identification. The cause of death was classified as per the International Classification of Diseases, CD10.

## 3. Statistical analysis

The statistical analysis was conducted using R Studio (4.2.0, US). We applied proper weights based on the NHANES complex sampling design to ensure that they were representative of the entire U.S. population. Continuous and categorical variables were expressed as the weighted average (standard error) and weighted percentages, respectively. The one-way analysis of variance (ANOVA) test and Kruskal Wallis test were used to analyze the statistical significance of the weighted categorical variables and continuous variables, respectively.

To evaluate all-cause mortality during follow-up, we constructed the cumulative Kaplan–Meier curves based on specific categories of grip strength and commodities. The log-rank test was used to determine whether differences in subgroups were statistically significant. The Cox-proportional risk model was used to analyze the relationship between grip strength, comorbidity, and all-cause mortality. Three different models were constructed using the multivariate Cox regression model: model 1 was adjusted for none; model 2 was adjusted for sex, age, and ethnic; and model 3 was adjusted for all covariates in model 2 along with education level, marital status, insurance status, PIR, BMI, waist circumference, smoking status, and alcohol consumption. The weighted generalized linear regression model was used to investigate the relationship between grip strength and comorbidity, and their linear relationship was discussed while controlling for covariables. A stratification analysis was conducted to examine the relationship between grip strength and all-cause mortality in different comorbidity groups and the interaction between grip strength and comorbidity with all-cause mortality. All statistical analyses had a two-sided *p*-value threshold for significance.

## 4. Results

Table 1 shows the weighted characteristics of the participants by tertile of grip strength. Overall, significant differences were noted in the age, sex, BMI, waist circumference, ethnic, marital status, education level, PIR, smoking status, alcohol consumption, and total comorbidities of the grip strength tertile groups (*p* < 0.05).

**Table 1:** Participants’ characteristics by tertile of grip strength.

Variable	Overall	Tertile 1	Tertile 2	Tertile 3	*p*-value
Age	69.46 (0.20)	72.61 (0.30)	68.44 (0.28)	67.57 (0.26)	<0.0001
Sex					<0.0001
Female	1,504 (50.81)	879 (93.82)	579 (69.56)	46 (3.45)	
Male	1,456 (49.19)	105 (6.18)	406 (30.44)	945 (96.55)	
BMI	28.80 (0.20)	28.06 (0.33)	29.20 (0.23)	29.07 (0.31)	0.02
Waist	102.01 (0.54)	97.32 (0.86)	101.51 (0.68)	106.36 (0.73)	<0.0001
Ethnic					< 0.0001
Black	716 (24.19)	156 (7.22)	240 (9.46)	320 (9.24)	
Mexican American	254 (8.58)	93 (4.21)	80 (3.24)	81 (2.91)	
White	1,400 (47.3)	506 (76.93)	457 (77.88)	437 (81.44)	
Other	590 (19.93)	229 (11.63)	208 (9.42)	153 (6.42)	
Marital					<0.0001
Divorced/separated	494 (16.71)	167 (13.78)	181 (18.40)	146 (9.55)	
Married/living with Partner	1,676 (56.68)	417 (49.84)	548 (61.35)	711 (80.61)	
Single	171 (5.78)	46 (3.49)	71 (4.53)	54 (4.44)	
Widowed	616 (20.83)	353 (32.89)	184 (15.72)	79 (5.40)	
Education					<0.0001
Above high school	1,444 (48.85)	419 (50.17)	508 (62.50)	517 (66.71)	
HIGH school or GED	686 (23.21)	233 (25.77)	225 (21.70)	228 (18.94)	
Less than high school	826 (27.94)	330 (24.06)	251 (15.80)	245 (14.36)	
PIR	3.08 (0.08)	2.57 (0.11)	3.13 (0.07)	3.47 (0.07)	<0.0001
Insurance status					0.37
No	245 (8.29)	63 (4.27)	96 (5.41)	86 (6.15)	
Yes	2,711 (91.71)	918 (95.73)	888 (94.59)	905 (93.85)	
Smoke					<0.0001
Former	1,108 (37.47)	288 (31.21)	354 (39.36)	466 (47.23)	
Never	1,466 (49.58)	602 (59.67)	499 (49.81)	365 (39.92)	
Now	383 (12.95)	93 (9.12)	131 (10.83)	159 (12.85)	
Alcoholic					<0.0001
No	934 (32.99)	459 (43.93)	312 (27.42)	163 (14.30)	
Yes	1897 (67.01)	468 (56.07)	630 (72.58)	799 (85.70)	
Comorbidities total					<0.0001
None	631 (21.32)	142 (14.22)	207 (20.25)	282 (26.19)	
One	822 (27.77)	250 (25.63)	285 (26.91)	287 (29.85)	
Two	644 (21.76)	217 (20.02)	234 (25.55)	193 (21.18)	
Three or more	863 (29.16)	375 (40.13)	259 (27.30)	229 (22.78)	

Continuous variables are expressed as weighted means (standard error), categorical variables as weighted percentages. BMI: body mass index; PIR: poverty income ratio.

**Figure 2:**
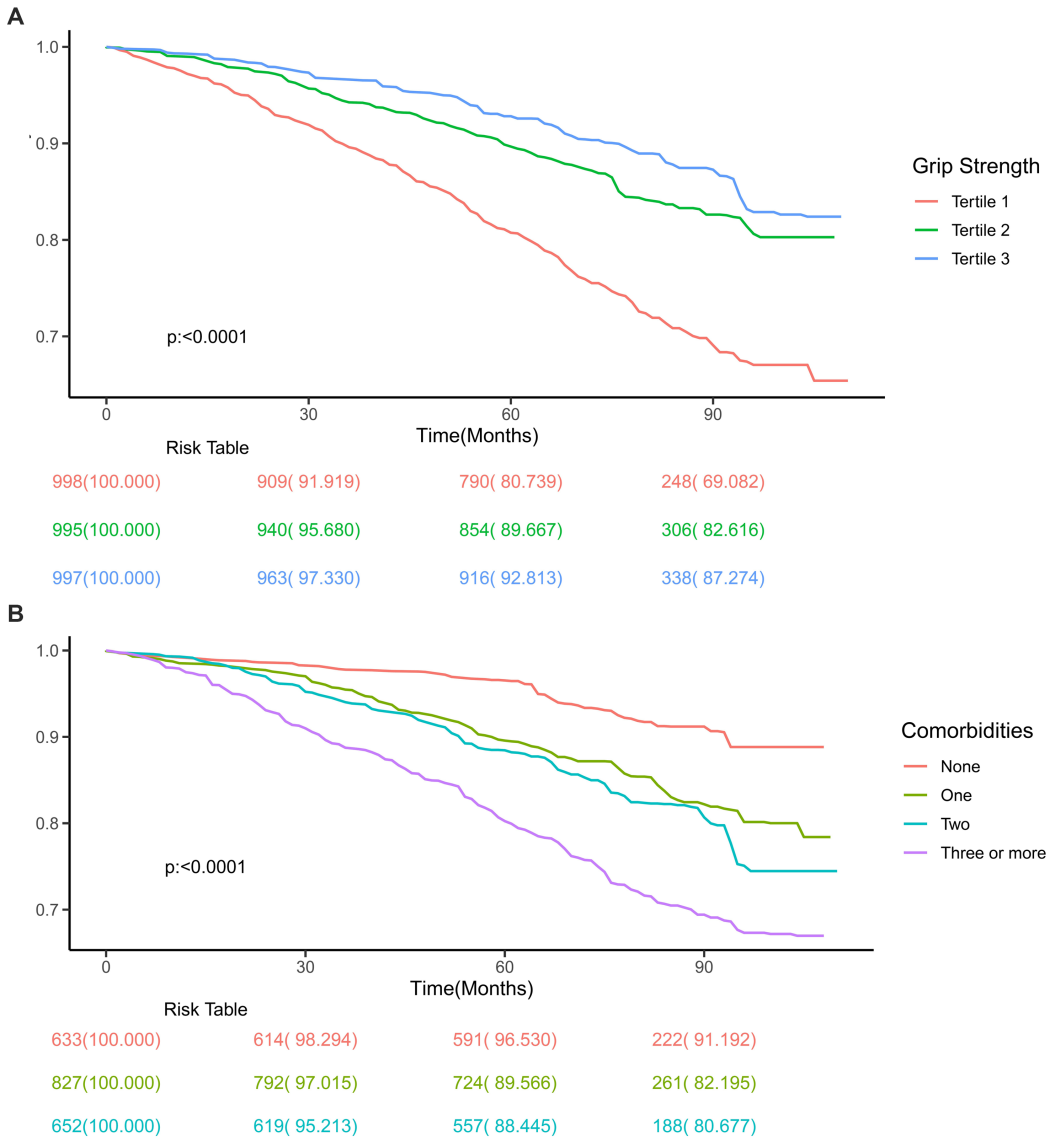
Association of grip strength **(A)** and comorbidities **(B)** with all-cause mortality, respectively.

Figure 2 depicts the survival curve of grip strength and comorbidity. Grip strength was inversely correlated with all-cause mortality, with a 5.8% reduction in the probability (*p* < 0.0001) of mortality risk for every 1 SD increase in grip strength. This negative correlation persisted even after multivariate correction (Table 2). When using grip strength was used as a categorical indicator from Cox regression estimates, the mortality risks in tertile 2 and tertile 3 groups decreased by 48.1% (95% CI, 0.427–0.632) and 60.2% (95% CI, 0.294–0.539), respectively, as compared to the overall risk of mortality in the tertile 1 group. This trend continued even after the multivariable adjustment analysis (Table 2). The risk of death increased gradually with increasing comorbidities in the model group irrespective of full adjustments (Table 2).

**Table 2:** Associations of the grip strength and Comorbidities with all-cause mortality, respectively.

	Model 1	Model 2	Model 3
Grip strength			
Per 1 SD increase	0.942 (0.930,0.955) <0.0001	0.916 (0.898,0.934) <0.0001	0.929 (0.906,0.952) <0.0001
Grip strength tertile			
Tertile 1	Ref	Ref	Ref
Tertile 2	0.519 (0.427,0.632) <0.0001	0.478 (0.364,0.626) <0.0001	0.528 (0.391,0.713) <0.001
Tertile 3	0.398 (0.294,0.539) <0.0001	0.226 (0.149,0.344) <0.0001	0.266 (0.168,0.419) <0.0001
*p* for trend	<0.0001	<0.0001	<0.0001
Comorbidities total			
None	Ref	Ref	Ref
One	1.968 (1.190,3.256) 0.008	1.449 (0.941,2.229) 0.092	1.827 (1.048,3.187) 0.034
Two	2.347 (1.475,3.736) <0.001	1.720 (1.112,2.659) 0.015	2.094 (1.217,3.602) 0.008
Three or more	3.806 (2.512,5.765) <0.0001	2.464 (1.644,3.695) <0.0001	2.677 (1.557,4.603) <0.001
*p* for trend	<0.0001	0.001	0.005

Model 1 was adjusted for none. Model 2 was adjusted for sex, age, and ethnic. Model 3 was adjusted for all covariates in model 2 and education, marital, insurance status, PIR, BMI, waist, smoke, alcoholic.

Table 3 shows the correlation between grip strength and comorbidities in the three weighted generalized models. Grip strength and multiple comorbidities had a significant linear negative correlation, but a significantly stronger negative correlation between grip strength and ≥3 comorbidities group (β = −2.219, −3.178~ −1.260, *p* < 0.001) was observed after multivariable adjustment (Model 3; Table 3).

**Table 3:** Weighted generalized linear regression analysis of the relationship between grip strength and Comorbidities.

	Model 1	Model 2	Model 3
None	Ref	Ref	Ref
One	−2.063 (−3.279,-0.847) 0.002	−0.599 (−1.521, 0.323) 0.193	−0.889 (−1.962, 0.184) 0.096
Two	−2.571 (−3.923,-1.218) <0.001	−0.509 (−1.367, 0.349) 0.233	−0.613 (−1.589, 0.362) 0.196
Three or more	−4.812 (−6.401,-3.223) <0.0001	−1.943 (−2.840,-1.045) <0.001	−2.219 (−3.178,-1.260) <0.001
*p* for trend	<0.0001	0.002	0.014

Model 1 was adjusted for none. Model 2 was adjusted for sex, age, and ethnic. Model 3 was adjusted for all covariates in model 2 and education, marital, insurance status, PIR, BMI, waist, smoke, alcoholic.

Table 4 shows the correlation between grip strength and mortality, stratified by comorbidities, using the lowest grip strength as the reference group. Grip strength and comorbidities showed no statistically significant associations with mortality. Interestingly, in individuals with ≥3 comorbidities, increasing grip strength decreased the likelihood of death (*p* for trend = 0.04).

**Table 4:** The association between grip strength and all-cause mortality by Comorbidities.

Grip strength tertile					
	Tertile 1	Tertile 2	Tertile 3	*p* for trend	*p* for interaction
Comorbidities					
None	Ref	0.99 (0.26, 3.75)	1.55 (0.25, 9.68)	0.56	0.45
One	Ref	0.57 (0.30,1.09)	0.25 (0.12,0.55)	0.09	
Two	Ref	0.62 (0.28,1.38)	0.47 (0.13,1.75)	0.24	
Three or more	Ref	0.54 (0.38,0.78)	0.22 (0.14,0.35)	0.04	

Analyses were adjusted for sex, age, ethnic, Education, marital, insurance status, PIR, BMI, waist, smoke, alcoholic.

## 5. Discussion

With an increase in the globally aging population, the health of the older adults is the most pressing issue in an aging society. From the standpoint of disease and function, comorbidity and disability are two of the crucial factors that affect the health of older people. The World Health Organization (WHO) released the International Classification of Functioning, Disability and Health (ICF), which is a framework that provides a detailed model for functional health and the relationship between disease symptoms and participation constraints (14). This study attempted to investigate the effects of two disease and functional status indicators, namely comorbidity and disability, on all-cause mortality in the older adults. Hypertension, which has the highest incidence among seniors, was selected as the indicator of the disease, and 15 self-reported chronic diseases were included as indicators of comorbidity; grip strength was chosen as the measurement index for assessing functional status. We, therefore, analyzed the relationship between grip strength and morbidities, and also studied their effects on all-cause mortality in an aged hypertensive population. The findings revealed that grip strength and comorbidities were related to all-cause mortality in an older hypertensive population, but the association of grip strength with mortality was not moderated by the number of comorbidities. The stratified analysis revealed that participants with more than three comorbidities who had higher grip strengths exhibited lower mortality rates.

Grip strength has a dynamic relationship with age. Grip strength is known to increase with age, peaking in adulthood. However, with increasing age, degenerative changes in body functions lead to a decline in grip strength. Our findings are consistent with previous research that found a link between grip strength and all-cause mortality, suggesting that decreased grip strength is associated with a higher risk of mortality (15–17). A meta-analysis of 38 studies involving 1,907,580 participants and 63,087 mortality cases used grip strength as a predictor for muscle strength and found that higher levels of grip strength were associated with a lower risk of all-cause mortality, where the association was slightly stronger in women (18). These results have been backed up by numerous prospective studies. For instance, a prospective cohort study which included data from the Survey of Health, Aging, and Retirement in Europe (2004–2017) revealed a negative correlation between CVD and grip strength in the middle-aged and older population (19). Similarly, a prospective study involving 502,293 participants aged 40–69 years from the UK Biobank discovered that higher all-cause mortality was associated with poor grip strength in both men and women. The majority of the participants in these studies, however, belonged to the general population (8, 16, 18). Our study provided evidence to support the association between grip strength and all-cause mortality in older people with hypertension. In this study, we used data from the NHANES database and found that grip strength was negatively associated with all-cause mortality in 2,990 hypertensive people aged >60. Here, grip strength was included as a continuous variable and the association was found after adjusting for hypertension (age, sex, race, BMI, and waist circumference), lifestyle (smoking and alcohol consumption), and socioeconomic (education, insurance, and PIR) -associated factors. After classifying grip strength into three groups, the older hypertensive patients in the low grip strength group were found to have a significantly lower survival rate than those in the high grip strength group. This suggests that grip strength can be used as a simple and practical predictor of survival in older hypertensive patients, and that increasing grip strength might lower all-cause mortality in older hypertensive patients.

This study proposed that grip strength is associated with mortality in older people with hypertension, where grip strength is a functional indicator that represents the intrinsic capacity of the older population. As the link between grip strength and comorbidity remains unknown, multiple studies have attempted to understand this association. According to Amy M.’s analysis of grip strength and comorbidity involving participants aged >50 years from the United States, grip strength gradually declined in adults with chronic diseases with multiple comorbidities, especially if the person had three or more chronic diseases (20). According to a cross-sectional study involving middle-aged and older community-dwelling adults using nationally representative data from six low- and middle-income countries, poor handgrip strength is associated with multiple chronic physical conditions and comorbidities (21). In a cross-sectional cohort of 1,145 subjects aged 50 and older from Hong Kong, multivariable-adjusted handgrip strength significantly decreased with the number of chronic diseases in men (*p* trend = 0.001) but had a marginally lesser effect in women (*p* trend = 0.06) (10). We are not aware of any studies that have been conducted on the relationship between grip strength and disease-specific comorbidity. In this study, the 15 self-reported chronic diseases from the database were chosen as comorbidity measures. In older people with hypertension, grip strength, and comorbidity were found to be linearly correlated. The presence of three or more diseases was significant after accounting for potential biological factors (including age, gender, and ethnicity), lifestyle choices, and socioeconomic factors.

There has been little research into whether functional and disease indicators have an impact on the survival of the older adults. This study did not find any interaction between the two parameters and the survival outcomes. However, stratified analysis revealed that grip strength had a stronger impact on the survival of patients with comorbidities, and enhancing grip strength could reduce mortality in hypertensive patients with more than three comorbidities. Rub’en et al. used marginal structural models (MSM) to provide a causal estimation of the association of hand grip strength with all-cause and cardiovascular mortality in a representative sample of adults aged 50 years or older. They found that these associations warranted promoting muscle-strengthening activities in adults and older adults, particularly those with pre-existing comorbidities (22). We, therefore, propose that older persons, especially those with multiple disorders, be encouraged to strengthen their muscle strength during exercise.

In the Global Report on Aging and Health, the WHO stated that healthy aging is the process of developing and maintaining the functions required for the older adults to live a healthy life. The synthesis of an individual’s intrinsic abilities and environmental characteristics, as well as their interaction, is defined as functional play. The five domains, namely locomotion, vitality, cognitive, psychological, and sensory can be used to evaluate these intrinsic abilities. Grip strength testing has proven useful for predicting physical and cognitive decline in older adults, both of which are crucial aspects of intrinsic capacity building (23). Furthermore, Ramirez-Velez R et al. demonstrated that the magnitude of grip strength significantly impacts intrinsic ability in the older adults. Poor grip strength in older people increases the risk of mental illness and contributes to cognitive decline, which reduces their intrinsic ability (24). Our next study will focus on investigating the correlation between grip strength and intrinsic ability in older people with hypertension.

This study has several limitations that are determined by its data source. First, because this was a cross-sectional study, it could not establish a direct causal link between grip strength, comorbidities, and all-cause mortality in older persons with hypertension. Second, because the comorbidity data for older hypertensive patients originated from a questionnaire and self-reported disorders, the accurate decision on the comorbidity status was biased. Thirdly, the inclusion criteria for the older hypertensive population did not allow for classification and risk stratification of the condition.

In conclusion, a significant linear correlation was found between grip strength and the number of comorbidities in the older hypertensive population without adjusting for control variables. Grip strength was negatively correlated with more than three comorbidities, even after adjusting for biological, lifestyle, and other factors, Comorbidity and all-cause mortality showed a positive correlation, while low grip strength was associated with decreased all-cause mortality. Although comorbidities had a greater impact on all-cause mortality, functional exercise appeared to improve the prognosis for patients with more than three diseases. This suggests that chronic disease prevention and appropriate treatment are necessary for older patients with multiple diseases and that functional exercise is assured to maintain good physical function. This lends credence to the concept of healthy aging, which is not always associated with diseases and their corresponding decline in functions.

## Data availability statement

Publicly available datasets were analyzed in this study. This data can be found at: https://www.cdc.gov/nchs/nhanes/index.htm.

## Ethics statement

The survey was conducted by the National Centers for Health’s Board of Approval Statistics and Institutional Review. Written informed consent for participation was not required for this study in accordance with the national legislation and the institutional requirements.

## Author contributions

YW, TM, and YR designed the study. YW and TM wrote the initial draft of the paper. YW and MY analyzed the statistics. XS, XW, and LC helped guide the writing. All authors contributed to the article and approved the submitted version.

## Conflict of interest

The authors declare that the research was conducted in the absence of any commercial or financial relationships that could be construed as a potential conflict of interest.

## Publisher’s note

All claims expressed in this article are solely those of the authors and do not necessarily represent those of their affiliated organizations, or those of the publisher, the editors and the reviewers. Any product that may be evaluated in this article, or claim that may be made by its manufacturer, is not guaranteed or endorsed by the publisher.

## References

[1] Global Burden of Disease Study C. Global, regional, and national incidence, prevalence, and years lived with disability for 301 acute and chronic diseases and injuries in 188 countries, 1990-2013: a systematic analysis for the global burden of disease study 2013. Lancet. (2015) 386:743–800. doi: 10.1016/S0140-6736(15)60692-4 26063472 PMC4561509

[2] Guiding principles for the care of older adults with multimorbidity: an approach for c. Guiding principles for the care of older adults with multimorbidity: an approach for clinicians: American Geriatrics Society expert panel on the Care of Older Adults with multimorbidity. J Am Geriatr Soc. (2012) 60:E1–E25. doi: 10.1111/j.1532-5415.2012.04188.x, PMID: 22994865 PMC4450364

[3] Schneider F Kaplan V Rodak R Battegay E Holzer B. Prevalence of multimorbidity in medical inpatients. Swiss Med Wkly. (2012) 142:w13533. doi: 10.4414/smw.2012.13533 22407848

[4] Dunlay SM Chamberlain AM. Multimorbidity in older patients with cardiovascular disease. Curr Cardiovasc Risk Rep. (2016) 10:8. doi: 10.1007/s12170-016-0491-8 27274775 PMC4889124

[5] Forman DE Maurer MS Boyd C Brindis R Salive ME Horne FM. Multimorbidity in older adults with cardiovascular disease. J Am Coll Cardiol. (2018) 71:2149–61. doi: 10.1016/j.jacc.2018.03.022, PMID: 29747836 PMC6028235

[6] Bohannon RW. Hand-grip dynamometry predicts future outcomes in aging adults. J Geriatr Phys Ther. (2008) 31:3–10. doi: 10.1519/00139143-200831010-00002, PMID: 18489802

[7] Celis-Morales CA Lyall DM Anderson J Iliodromiti S Fan Y Ntuk UE. The association between physical activity and risk of mortality is modulated by grip strength and cardiorespiratory fitness: evidence from 498 135 UK-biobank participants. Eur Heart J. (2017) 38:116–22. doi: 10.1093/eurheartj/ehw249, PMID: 28158566 PMC5837781

[8] Prasitsiriphon O Pothisiri W. Associations of grip strength and change in grip strength with all-cause and cardiovascular mortality in a European older population. Clin Med Insights Cardiol. (2018) 12:117954681877189. doi: 10.1177/1179546818771894 PMC5987902 29881318

[9] Wang YC Liang CK Hsu YH Peng LN Chu CS Liao MC. Synergistic effect of low handgrip strength and malnutrition on 4-year all-cause mortality in older males: a prospective longitudinal cohort study. Arch Gerontol Geriatr. (2019) 83:217–22. doi: 10.1016/j.archger.2019.05.007, PMID: 31100544

[10] Cheung CL Nguyen US Au E Tan KC Kung AW. Association of handgrip strength with chronic diseases and multimorbidity: a cross-sectional study. Age (Dordr). (2013) 35:929–41. doi: 10.1007/s11357-012-9385-y, PMID: 22314403 PMC3636411

[11] Volaklis KA Halle M Thorand B Peters A Ladwig KH Schulz H. Handgrip strength is inversely and independently associated with multimorbidity among older women: results from the KORA-age study. Eur J Intern Med. (2016) 31:35–40. doi: 10.1016/j.ejim.2016.04.001, PMID: 27108239

[12] Curtin LR Mohadjer LK Dohrmann SM Kruszon-Moran D Mirel LB Carroll MD. National Health and nutrition examination survey: sample design, 2007-2010. Vital Health Stat 2. (2013) 2013:1–23. 25090039

[13] Johnson CL Paulose-Ram R Ogden CL Carroll MD Kruszon-Moran D Dohrmann SM. National health and nutrition examination survey: analytic guidelines, 1999-2010. Vital Health Stat 2. (2013) 2013:1–24. 25090154

[14] WHO. International classification of functioning, disability and health: ICF; WHO Geneva, Switzerland. (2001).

[15] Laukkanen JA Voutilainen A Kurl S Araujo CGS Jae SY Kunutsor SK. Handgrip strength is inversely associated with fatal cardiovascular and all-cause mortality events. Ann Med. (2020) 52:109–19. doi: 10.1080/07853890.2020.1748220, PMID: 32223654 PMC7877981

[16] Yates T Zaccardi F Dhalwani NN Davies MJ Bakrania K Celis-Morales CA. Association of walking pace and handgrip strength with all-cause, cardiovascular, and cancer mortality: a UK biobank observational study. Eur Heart J. (2017) 38:3232–40. doi: 10.1093/eurheartj/ehx449, PMID: 29020281 PMC5837337

[17] Cai Y Liu L Wang J Gao Y Guo Z Ping Z. Linear association between grip strength and all-cause mortality among the elderly: results from the SHARE study. Aging Clin Exp Res. (2021) 33:933–41. doi: 10.1007/s40520-020-01614-z, PMID: 32524391

[18] Garcia-Hermoso A Cavero-Redondo I Ramirez-Velez R Ruiz JR Ortega FB Lee DC. Muscular strength as a predictor of all-cause mortality in an apparently healthy population: a systematic review and Meta-analysis of data from approximately 2 million men and women. Arch Phys Med Rehabil. (2018) 99:2100–2113.e5. doi: 10.1016/j.apmr.2018.01.008, PMID: 29425700

[19] Peralta M Dias CM Marques A Henriques-Neto D Sousa-Uva M. Longitudinal association between grip strength and the risk of heart diseases among European middle-aged and older adults. Exp Gerontol. (2022) 171:112014. doi: 10.1016/j.exger.2022.112014 36347359

[20] Yorke AM Curtis AB Shoemaker M Vangsnes E. The impact of multimorbidity on grip strength in adults age 50 and older: data from the health and retirement survey (HRS). Arch Gerontol Geriatr. (2017) 72:164–8. doi: 10.1016/j.archger.2017.05.011, PMID: 28667843

[21] Vancampfort D Stubbs B Firth J Koyanagi A. Handgrip strength, chronic physical conditions and physical multimorbidity in middle-aged and older adults in six low- and middle income countries. Eur J Intern Med. (2019) 61:96–102. doi: 10.1016/j.ejim.2018.11.007, PMID: 30509483

[22] Lopez-Bueno R Andersen LL Calatayud J Casana J Smith L Jacob L. Longitudinal association of handgrip strength with all-cause and cardiovascular mortality in older adults using a causal framework. Exp Gerontol. (2022) 168:111951. doi: 10.1016/j.exger.2022.111951, PMID: 36096322

[23] Rijk JM Roos PR Deckx L van den Akker M Buntinx F. Prognostic value of handgrip strength in people aged 60 years and older: a systematic review and meta-analysis. Geriatr Gerontol Int. (2016) 16:5–20. doi: 10.1111/ggi.12508, PMID: 26016893

[24] Ramirez-Velez R Correa-Bautista JE Garcia-Hermoso A Cano CA Izquierdo M. Reference values for handgrip strength and their association with intrinsic capacity domains among older adults. J Cachexia Sarcopenia Muscle. (2019) 85:278–86. doi: 10.1016/S0065-2113(04)85005-3 PMC6463468 30843369

